# Antimicrobial Resistance Associated with Mass Gatherings: A Systematic Review

**DOI:** 10.3390/tropicalmed10010002

**Published:** 2024-12-24

**Authors:** Linda Tong Pao, Mohamed Tashani, Catherine King, Harunor Rashid, Ameneh Khatami

**Affiliations:** 1 Sydney Children’s Hospitals Network, Westmead, NSW 2145, Australia; linda.tongpao@health.nsw.gov.au (L.T.P.); ameneh.khatami@health.nsw.gov.au (A.K.); 2 The Children’s Hospital at Westmead Clinical School, Faculty of Medicine and Health, The University of Sydney, Westmead, NSW 2145, Australia; mohamed.tashani@health.nsw.gov.au; 3 Discipline of Paediatrics & Child Health, The University of New South Wales, Kensington, NSW 2033, Australia; 4 Department of Paediatrics, Faculty of Medicine, University of Tripoli, Tripoli 13275, Libya; 5 Sydney Infectious Diseases Institute, The University of Sydney, Westmead, NSW 2145, Australia; 6 Sydney School of Public Health, The University of Sydney, Sydney, NSW 2006, Australia

**Keywords:** antibiotics, antimicrobial resistance, Arbaeen, Hajj, systematic review, mass gathering, Umrah

## Abstract

Mass gatherings are associated with the spread of communicable diseases. Some studies have suggested that acquisition of antimicrobial resistance (AMR) may be associated with attendance at specific mass gatherings. This systematic review aimed to synthesise evidence on the association between attendance at mass gatherings and antimicrobial resistance (AMR) and assess the prevalence of AMR at mass gatherings. A literature search of the Cochrane, Medline, Scopus, and Embase databases was performed. Studies were included if they reported original data, involved mass gatherings, and reported AMR results. Of 5559 titles screened, 44 studies met the inclusion criteria, most of which (*n* = 40) involved religious mass gatherings. The heterogeneity of the studies precluded a meta-analysis, hence a narrative synthesis by organism was conducted. A significant increase in antibiotic-resistant *Escherichia coli* and *Klebsiella pneumoniae* was reported following Hajj, as was a rise in gastrointestinal carriage of extended-spectrum β-lactamase (ESBL) or carbapenemase genes. Carriage of *Streptococcus pneumoniae* isolates non-susceptible to one or more antibiotics was also shown to increase from pre-Hajj to post-Hajj. There appears to be an association between attendance at mass gatherings and the acquisition of some AMR phenotypes and genotypes in some significant human pathogens, including *E. coli* and *S. pneumoniae*.

## 1. Introduction

Although most human pathogens have inherent resistance mechanisms against a range of antimicrobials, global concern about increasing antimicrobial resistance (AMR) generally refers to genetic changes in clinically significant bacteria that were previously susceptible to a particular antibiotic to become resistant. These can be due to random mutations or the acquisition of intact gene(s) associated with AMR. The drivers for these changes are multifactorial but include selection pressure introduced by antimicrobial use in humans, animals, and agriculture [1,2]. In bacteria, resistance genes can be found on both chromosomal and transmissible extrachromosomal genetic elements [1]. Once present, AMR genes can be passed on by clonal replication or spread to other bacteria through horizontal gene transfer [2]. Person-to-person spread through close contact is one of the key mechanisms driving the community spread of AMR genes, which includes mass gathering settings [3,4,5].

Mass gatherings present a unique problem for the transmission of infectious diseases. The World Health Organization (WHO) defines a mass gathering as an event in which the number of attendees exhausts the resources of the community or country hosting the event [6]. Traditionally, mass gatherings are considered gatherings of more than one thousand people and include events such as the Olympic Games, Hajj pilgrimage, and other major sporting, religious, and cultural events [7]. Such events pose a significant and unique public health problem with the possibility of the emergence and/or spread of novel infections and AMR, as suggested by reports of increasing detection of drug-resistant organisms in environmental water samples, such as ESBL-positive *Escherichia coli* in settings of Hajj [8], the Olympic Games [9], and Kumbh Mela [10].

Previously published scoping reviews have investigated the association between mass gatherings and AMR with a focus on specific AMR organisms or specific mass gatherings [4,11,12]. To our knowledge, a systematic review to collate the published literature on AMR related to any micro-organism in the context of mass gatherings has not been performed to date. The objective of this study was, therefore, to synthesise evidence on the association between attendance at mass gatherings and antimicrobial resistance (AMR) by examining the frequency and pattern of AMR reported across various study designs.

## 2. Materials and Methods

### 2.1. Search Strategy

This systematic review was prospectively submitted for registration on PROSPERO (Registration number: CRD42019145118), and PRISMA reporting guidelines have been adhered to [13,14].

Literature searches were completed in key bibliographic databases to locate publications on AMR associated with mass gatherings. The following databases were searched by an experienced information specialist (CK): OVID Medline All, including Epub Ahead of Print; In-Process and Other Non-Indexed Citations; Daily and Versions (1946-18 November 2024); OVID Embase (1974-19 November 2024); Cochrane Library Database of Systematic Reviews (Issue 11 of 12, November 2024); Cochrane Library Central Register of Controlled Trials (Issue 10 of 12, October 2024); and SCOPUS (1823-21 November 2024). Where possible, a combination of both database-controlled vocabulary and equivalent text-word terms was used. These terms included ‘Drug Resistance, Microbial’, ‘Anti-Bacterial Agents’, ‘Anniversaries and Special Events’, and ‘Crowding’. These were supplemented with a range of text-word terms for multi-drug resistance, different types of mass gatherings, specific religious events such as Hajj, Umrah, Kumbh Mela, and specific sporting events such as the Olympics, Commonwealth Games, and World Cup. In the scoping search phase for the review, the MeSH terms used to index key mass-gathering articles were carefully examined to locate appropriate MeSH descriptors. The very broad MeSH term ‘Anniversaries and Special Events’ was chosen as it includes any ‘occasions to commemorate an event or occasions designated for a specific purpose’. Likewise, the use of the exploded form of the MeSH term ‘Crowding’ includes narrower MeSH terms such as ‘Mass Gatherings’ and relates to crowding in any context. The use of broad, truncated text words such as ‘crowd$.tw’, ‘pilgrim$.tw’, ‘tournament$.tw’ and ((music$ or religio$ or cultural$) adj2 festival$).tw also aims to capture multiple potential aspects related to mass gatherings. Similarly, the use of ‘(mass adj2 gather$).tw’ is designed to capture any variant title or abstract use of the mass gathering term. No date or language limits were used. The last search was conducted on 21 November 2024. The full search strategies, including all terms used, are provided in a Supplementary File (Box S1).

### 2.2. Inclusion and Exclusion Criteria

Articles were included if (a) they were published in English, (b) the study reported original data, (c) they were set within the context of a mass gathering of at least 1000 individuals, and (d) reported any AMR results, even if the result was zero. Manuscripts were excluded if they (a) were set in closed environments such as military barracks, hospitals, schools, or universities or reported results on only environmental samples, and (b) were cross-sectional surveillance studies that included a mass gathering event but did not specifically report results for the event.

### 2.3. Screening, Abstraction, and Quality Assessment

An initial screen of all manuscripts identified by the literature search was performed by the first author (LTP) based on title and abstract review and cross-checked by a second author (AK or HR) to identify articles that potentially met inclusion or exclusion criteria. Authors (AK and HR) then identified the final list of studies that met all inclusion and exclusion criteria after a full-text review of manuscripts. The reference lists of included studies and other relevant reviews were scrutinised to identify any other articles that were not picked up in the initial literature search. Data extraction from all included studies was performed independently by two authors (initially by LTP and AK, subsequently by MT and HR). Any discrepancies were resolved by discussion between all authors. The primary outcome of interest was the new acquisition of an AMR organism or gene in attendees of any mass gathering (forcibly displaced people dwelling in communities were not considered a mass gathering for this review).

Secondary outcomes included the overall prevalence of specific AMR organisms (i.e., the prevalence of carriage or infection with infective agents that exhibit AMR) or genes documented at or after attendance at a mass gathering. Where possible, data extracted from multiple studies with similar methodologies (for example, the same organism of interest, same resistance profile, and similar detection methods) were aggregated to arrive at an overall crude estimate of prevalence.

The following information was extracted from included studies to the extent available either through the published manuscript or through direct contact with the corresponding author: study year(s); study type; mass gathering setting (including country); study setting (e.g., community, clinic, hospital); study objectives; before/after sample sizes (including individuals and clinical samples); sample type and sampling method; proportion male; age distribution; inclusion of travellers or locals; countries or ethnicities of participants; comorbidities; any comparative group; clinical syndrome investigated (e.g., infectious syndrome or colonisation); organism of interest; number of each organism identified; laboratory methods for identification of organism and resistance mechanisms; phenotypic resistance identified; resistance genes identified; antibiotic exposures identified; other interventions; other outcome measures; study limitations and funding source. The Newcastle–Ottawa Scale (NOS) was used to assess the quality of included studies [15].

## 3. Results

### 3.1. General Description of Included Studies and Quality Assessment

**Figure 1:**
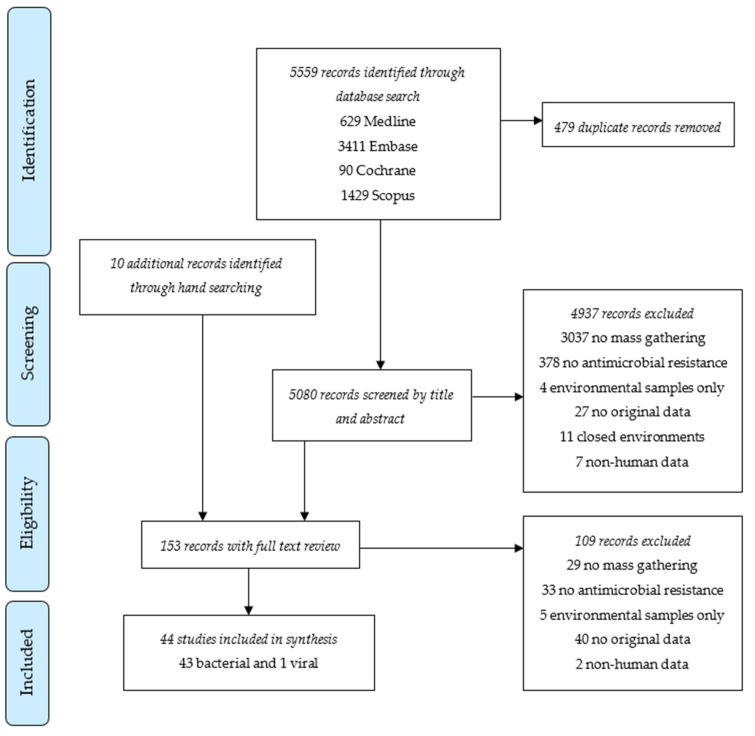
PRISMA flow chart of study selection for systematic review.

Forty-four studies were included in the systematic review (Figure 1). All were observational studies with 17 cross-sectional studies, 16 longitudinal cohort studies, five case series or case reports, and six other types, such as outbreak investigations and retrospective case note reviews (Table 1). Among these, 36 studies involved Hajj and/or Umrah pilgrimages, three involved entertainment events, two involved Arbaeen pilgrimages, another two involved Grand Magal de Touba, and one involved a political protest. Except for one study that evaluated the existence of oseltamivir-resistant influenza A(H1N1)pdm09 viruses [16], all included studies described AMR in bacterial pathogens.

The studies were clinically highly heterogeneous due to diverse study designs, settings, laboratory methods applied, and pathogens explored. Thus, only a narrative synthesis by organism was derived and a meta-analysis or aggregation was not attempted.

Where possible, data extracted from multiple studies with similar methodologies (for example, the same organism of interest, same resistance profile, and similar detection methods) were aggregated to arrive at an overall crude estimate of point prevalence.

Excluding five case reports/series, all the remaining 39 observational studies were assessed according to the NOS guidelines for the quality of studies in research articles. The NOS scores of individual studies are provided in Table 1. Two manuscripts scored 7, nine scored 6, 10 scored 5, 18 scored < 5, and the other five were not scored (due to being case reports/series), indicating most studies were below average quality. Out of 39 studies that were assessed using the NOS scale, 29 studies scored a point in ‘representativeness of exposed cohort domain’, four in ‘selection of the non-exposed cohort’, 31 in ‘ascertainment of exposure’, 28 in ‘outcome of interest not being present at start of the study’, two in ‘comparability of cohorts’, 37 in ‘assessment of outcome’, 19 in ‘long enough follow up’ and another 19 in ‘adequacy of follow up’. This indicates most studies did not have a non-exposed cohort to compare.

The laboratory diagnostic methods and statistical tests used in individual studies and summary findings by mass gathering category have been provided in Table 2.

### 3.2. Escherichia coli

Table 3 and Table 4 outline the 11 studies that reported AMR in *E. coli* isolates. A total of 2812 samples were collected from 2881 participants enrolled across these studies, with 482 *E. coli* isolates identified from rectal swabs, faeces, urine, sputum, or skin swabs.

Five longitudinal cohort studies reported results based on screening of either rectal and/or pharyngeal swabs in otherwise well individuals (Table 3). Leangapichart et al. demonstrated a statistically significant increase in carriage of antibiotic-resistant *E. coli* identified by phenotypic antibiotic susceptibility testing (AST) among pilgrims after attendance at Hajj compared to pre-Hajj rectal swabs (ceftriaxone-resistant *E. coli* 3.9% [5/129] to 14% [18/129], *p* = 0.008; ticarcillin-clavulanic acid-resistant *E. coli* 12.4% [16/129] to 22.5% [29/129], *p* = 0.048, *E. coli* resistant to any of six antibiotics tested 14% [18/129] to 28% [36/129], *p* = 0.009). This study also demonstrated a significant increase in the acquisition of CTX-M genes with 10.1% (13/129) of pre-Hajj samples and in 32. 6% (42/129) post-Hajj samples (*p* < 0.001) identified the gene [32]. Two other studies performed by the same group also showed a similar increase in the prevalence of this resistance from before to after Hajj [33,34]. There was also a statistically significant increase in carriage of the *mcr-1* gene associated with colistin resistance across two Hajj seasons (from 1–2% to 8–9%, *p* ≤ 0.01) [34]. In another cohort study conducted by Hoang and colleagues, *E. coli* was isolated from the rectal specimens of around 5% of French pilgrims before Hajj and around 10% after Hajj, with different distributions of resistance genes before and after Hajj [50]. Finally, one study explored AMR patterns in paired rectal swabs collected from 290 domestic pilgrims before and after attending the Grand Magal de Touba in Senegal. Sixty *E. coli* strains were identified by culture, with nearly all resistant to amoxicillin and ceftriaxone but sensitive to carbapenems. One hundred and five participants (36.2%) acquired at least one resistance gene detected using culture-independent molecular methods, notably CTX-M A (21.0%), SHV (16.5%), and TEM (8.2%) [58].

Two cross-sectional studies also documented ESBL or carbapenemase resistance genes in *E. coli* isolates identified from pilgrims attending Hajj [17,18]. Another cross-sectional study described multidrug-resistant (MDR) *E. coli* among attendees of El-Tahrir Square Protest in Cairo in 2011. Four combinations of six resistance genes (tet[A], dhfrI, dhfrV, dhfrXIII, sulI, and sulII) were seen in around two-thirds of strains [41]. Finally, two other cross-sectional studies reported just phenotypic antibiotic resistance among Umrah pilgrims and attendees of the Glastonbury music festival [24,30].

Not all studies provided numerators and denominators for isolates tested, so a precise estimation was not possible; however, roughly across all included studies, phenotypically detected resistance among *E. coli* isolates identified from attendees of any mass gathering occurred in 16.1% (61/380) to trimethoprim-sulfamethoxazole, 27.1% (103/380) to ciprofloxacin, 36.8% (140/380) to third-generation cephalosporins, and 4.5% (17/380) to aminoglycosides.

### 3.3. Klebsiella pneumoniae

Nine studies reported AMR among *Klebsiella* species (Table 5), including four longitudinal studies [32,34,50,58], four cross-sectional studies [19,36,52,55], and one retrospective audit of hospital records [30]. Not all studies provided numerators and denominators, and antibiotics tested were diverse; however, overall, phenotypically detected resistance among *K. pneumoniae* isolates identified from attendees of Hajj and Umrah mass gatherings occurred in 95.8% (92/96) to ampicillin, 50.7% (35/69) to amoxycillin–clavulanate, 19.7% (39/198) to third-generation cephalosporins, and 14.6% (28/192) to gentamicin. The three longitudinal studies conducted at Hajj demonstrated an increase in or acquisition of resistance genes, notably blaCTX-M-15 (+TEM and SHV genes), blaCTX-M-14, blaSHV-161, mcr1, and blaTEM-1 [32,34,50]. The only longitudinal study conducted at Grand Magal de Touba identified only two strains of *K. pneumoniae* (one before and the other after attending the mass gathering); both were susceptible to carbapenems and aminoglycosides, but were resistant or intermediately susceptible to amoxicillin [58].

### 3.4. Organisms Carrying ESBL Genes

Table 6 outlines studies reporting the carriage of ESBL genes detected by culture-independent molecular assays. Overall, carriage of CTX-M genes occurred at a rate of 8.0% (49/615) to 25.70% (158/615).

### 3.5. Staphylococcus aureus

Among twelve articles that reported AMR in *S. aureus*, six were cross-sectional studies, four were longitudinal cohort studies, and the other two were retrospective case note reviews (Table 7). A total of 5217 participants were enrolled in these studies, from whom 8106 samples were collected (skin swabs, secretions, sputum, blood, or urine). *S. aureus* was identified in 1736 samples collected. MRSA was identified in 22.5% (390/1736) through phenotypic AST. In addition, 85.6% (632/738) of *S. aureus* isolates were resistant to penicillin, 13.2% (101/764) to clindamycin, and 6.2% (45/731) to trimethoprim–sulfamethoxazole. The overall prevalence of MRSA genes detected in five studies that evaluated the presence of resistance genes was 27.1% (175/645). Of three longitudinal studies set during Hajj, only one demonstrated a significant increase in rates of MRSA isolates detected amongst all *S. aureus* isolates (4.1% [25/606] versus 10.6% [62/606]) [59]. The only study that determined nasopharyngeal carriage of MRSA at Grand Magal de Touba did not report an increase in MRSA colonisation following attendance at the mass gathering (5.2% vs. 2.6%) [57].

### 3.6. Streptococcus Species

Eight studies described AMR in *Streptococcus* species (predominantly *S. pneumoniae* in seven studies), including five cross-sectional studies, two prospective longitudinal studies, and one retrospective case note review (Table 8). A total of 7860 participants were enrolled in these studies with 10,299 samples collected (sputum, bronchoalveolar lavage, naso-, or oropharyngeal swabs). A total of 672 *Streptococcus* species were identified, of which the majority, 93.5% (628/672), were *S. pneumoniae*; 3.6% (24/672) isolates were *S. pyogenes*, and there were 3.0% (20/672) uncharacterised *Streptococcus* species. Three studies that compared phenotypic antibiotic resistance of *S. pneumoniae* among pilgrims before and after Hajj demonstrated that overall pneumococcal carriage increased post-Hajj from 9.1% (294/3210) to 11.2% (364/3233), *p* < 0.01 [27,37,53]. Penicillin non-susceptible isolates occurred among 21.5% (109/507) *S. pneumoniae* isolates, and in 0.9% (3/348) were classified as non-susceptible to third-generation cephalosporins.

### 3.7. Salmonella and Other Bacteria

One case report documented enteric fever in a pilgrim returning to London from the Arbaeen pilgrimage in Iraq in 2019. The patient had Salmonella enterica serovar Typhi isolated from a blood culture with the strain phenotypically confirmed to be an ESBL producer, harbouring the blaCTX-M-15 resistance gene. It was also resistant to fluoroquinolones with mutations in the gyrA quinolone resistance-determining region [28]. A second report documented a series of patients in the US with genetically linked isolates of S. Typhi with the same resistance pattern (ceftriaxone-resistant) following travel to Iraq, of which four cases were temporally associated with the Arbaeen pilgrimage, although no specific details regarding attendance at Arbaeen was documented [26].

A case of pneumonia caused by MDR *E. americana* was reported in a young Indonesian pilgrim admitted to a Makkah hospital following a severe road traffic accident. The isolated bacterium was resistant to most commonly used antibiotics, including third-generation cephalosporins and aminoglycosides [23]. In a cross-sectional study involving 129 Hajj pilgrims during Hajj 2018, Bokhary et al. identified six *H. influenzae* strains that were resistant to ampicillin. Some were additionally resistant to other antibiotics, including one to cefuroxime, cefotaxime, and cefepime, another to cefuroxime, and two others to trimethoprim–sulfamethoxazole [48].

There were three reports of meningococcal isolates exhibiting resistance to ciprofloxacin associated with Hajj and Umrah pilgrimages. A cross-sectional study involving 616 Umrah pilgrims reported three meningococcal carriage strains, one of which (serogroup B) exhibited resistance to ciprofloxacin and intermediate susceptibility to trimethoprim-sulfamethoxazole but was sensitive to other antibiotics, including penicillin [49]. A subsequent longitudinal carriage study recruiting 3921 pilgrims before and after the Hajj 2019 described 0.7% (58 out of 7842 swabs) as positive for meningococcus, and 94.8% (55/58) of those were resistant to ciprofloxacin but sensitive to benzyl penicillin [56]. The same year, Public Health England reported three cases of meningococcal conjunctivitis (by non-groupable strains) among pilgrims with contact after the Hajj 2019 that were resistant to ciprofloxacin and with intermediate susceptibility to penicillin [22]. Finally, a prospective study that evaluated antimicrobial sensitivity of meningococci among Hajj and Umrah pilgrims with meningococcal infection in 1992–1993 reported that the isolates were sensitive to most of the antibiotics that were commonly used to treat meningococcal infection during that era, including penicillin and chloramphenicol, but only 50% (7/14) were sensitive to cotrimoxazole and 71% (10/14) to tetracycline [45].

### 3.8. Tuberculosis

From sputum collected from 1164 Hajj pilgrims in 2015, 1.4% (15/1063) for whom Xpert MTB/RIF^®^ assay results were available were identified as having undiagnosed tuberculosis (TB). No rpoB gene mutations (associated with rifampicin-resistance) were detected, and phenotypic resistance was not reported [46]. A larger study conducted during the Hajj in 2016 and 2017 similarly identified 0.7% (10/1510) of non-hospitalised patients as positive for TB, with no rifampicin resistance detected. In contrast, among 2.9% (9/304) of hospitalised pilgrims who were positive for TB, two also tested positive for rifampicin resistance [54].

### 3.9. Influenza

One study that reported on influenza A(H1N1)pdm09 met inclusion criteria. Among 305 pilgrims attending Hajj, influenza A(H1N1)pdm09 was detected in five (1.6%) pharyngeal swabs collected as they returned to their home country of Iran. All strains were sensitive to oseltamivir and did not carry the H275Y resistance mutation [16].

## 4. Discussion

Mass gatherings can see one thousand to several million travellers from different countries across the globe congregate in one area for a specified amount of time. This sets up a unique opportunity for the emergence and transmission of novel pathogens as well as AMR. With the rise of AMR as one of the greatest threats to global health in the 21st century, attendance at mass gatherings may represent an important risk. This review demonstrates that mass gatherings appear to play a contributory role in the acquisition and worldwide spread of AMR, for at least some pathogens.

Among the studies that met inclusion criteria for this systematic review, the most robust data exist for AMR in *E. coli* isolates identified from pilgrims attending Hajj. Phenotypically detected resistance in *E. coli* carriage isolates has been shown to increase between pre- and post-Hajj cohorts, with statistically significant increases in resistance to third-generation cephalosporins [32]. A significant increase in the prevalence of ESBL genes (CTX-M genes) has also been demonstrated among *E. coli* carriage isolates identified in pilgrims from pre-Hajj to post-Hajj. Additionally, in culture-independent molecular assays of gastrointestinal specimens, the identification of CTX-M genes also increased, with the acquisition of CTX-M genes demonstrated in almost one-third of individuals. These findings are consistent with results from Tängdèn et al., who demonstrated prospectively for the first time acquisition of these resistance genes after international travel. In culture-independent molecular assays of rectal swabs, 24% of Swedish travellers with negative pre-travel samples were colonised with ESBL-producing *E. coli* after travel outside Northern Europe. All strains carried CTX-M genes, the majority of which were blaCTX-M-15. Acquisition of ESBL-producing *E. coli* occurred in 88% of travellers to India, 32% of travellers to other parts of Asia, 29% of travellers to the Middle East, 13% after travel to Southern Europe, and 4% after travel to Africa [60]. Of note, all isolates from India were blaCTX-M-15 positive, which correlated with the prevalent ESBL gene documented in India at the time [61].

Geographical differences in ESBL-producing *E. coli* are widely reported, and the high prevalence of blaCTX-M-15 detected in samples from Hajj pilgrims in this review is probably reflective of the worldwide spread of these genes. The prevalence of ESBL-producing *E. coli* has been reported in the Saudi Arabian human population, environmental samples, and animals in the country as 16.6–36.4% [11,62]. During the 2019 Hajj, 58% of pilgrims were from Asian countries, 27% were from Arab countries, 9% were from African countries, 4.8% were from European countries, and 1.4% were from North and South America and Australia [63]. Thus, Hajj attendees travel from countries that have variable prevalence of ESBL-producing *E. coli* [64]. This demonstrates that this, in combination with other factors such as the ubiquitous nature of ESBL genes, their ease of transmissibility, as well as crowded living conditions during Hajj, may contribute to individual acquisition of these genes by pilgrims. Countries with lower national prevalence, such as Sweden, are therefore particularly at risk when residents travel to an area with a higher prevalence of AMR or attend mass gatherings such as Hajj.

In contrast, data relating to *S. aureus* isolated from attendees at mass gatherings such as Hajj do not demonstrate a higher rate of AMR than that seen in the general population. Average MRSA rates of 38% have been reported in Saudi Arabia, with some regional variability [65], which suggests that the highest rates seen in the Western region of Saudi Arabia may be attributed to the presence of holy sites visited by pilgrims each year; however, the results from this review do not support this. Overall, around 21% of *S. aureus* isolates among Hajj pilgrims have been phenotypically or genotypically identified as MRSA. Rates of resistance to penicillin (85.6%), clindamycin (13.2%), and trimethoprim–sulfamethoxazole (6.2%) are also very similar to data from Australia and other relatively low-incidence countries. The Australian report on antimicrobial use and resistance in human health reported the national prevalence of MRSA in 2020–2022 as 15–19% among *S. aureus* isolates identified from blood and other specimens, with 81–85% of isolates resistant to penicillin [66]. Globally, there is variability in rates of MRSA [67]; however, in the only longitudinal study conducted to date, increased rates of MRSA carriage were not seen in attendees of mass gatherings, specifically Hajj [59].

The genes encoding the production of ESBL enzymes and the mecA gene can be transmitted by horizontal gene transfer [68]; however, the ubiquitous nature of ESBL genes and the transmissibility of the plasmids on which they are often found [69] likely contributes to higher rates of acquisition compared to MRSA. MRSA is transmitted between individuals by contaminated hands and direct contact with a colonised or infected person [70]. In contrast, Enterobacteriaceae thrive in the gastrointestinal tract and, when shed, can be found on contaminated hands, surfaces, food, and water, allowing acquisition from consumption of contaminated food or water. Additionally, ESBL genes are easily transferred between different Enterobacteriaceae species, including those found widely in the environment [71].

For *S. pneumoniae*, the overall carriage was shown to increase significantly from 9.1% to 11.2%, with an increase in carriage of isolates non-susceptible to one or more antibiotics from pre-Hajj to post-Hajj samples [27,37,53]. Increased phenotypic diversity in post-Hajj isolates was also reported, with differences in the allelic profile of MDR strains of *S. pneumoniae* between pre- and post-Hajj isolates indicating that attendance at Hajj favours the acquisition of new strains with a higher rate of resistance [27]. This provides support for the recommendations from the Saudi Thoracic Society and the American Advisory Committee on Immunisation Practices for pneumococcal vaccination to be administered to Hajj pilgrims at higher risk for pneumococcal disease, e.g., those aged over 50 years [72]. There are other vaccines also recommended for travellers planning to attend mass gatherings [73,74]. The role of vaccinations in reducing the reliance on antibiotics and downstream effects on AMR is well recognised [75]. Vaccines against diseases such as meningococcal and pneumococcal diseases, COVID-19, and influenza are recommended and sometimes mandatory, depending on the travel destination. For example, COVID-19 and meningococcal vaccines are mandatory for attendance at the Hajj, whereas seasonal influenza and pneumococcal vaccines are strongly recommended [76]. Similarly, vaccinations against measles, hepatitis A and B, and in some regions, yellow fever and typhoid, are advised based on the local epidemiology and specific activities during the event [73,77]. However, vaccinations are often underutilised, and travellers often miss even mandatory vaccines [78]. There is thus room for improvement in global immunisation strategies for mass gatherings, including broader access to vaccines, enhanced public health campaigns to promote pre-travel vaccinations, and the development of tailored recommendations for emerging pathogens. The involvement of tour operators in vaccination advice is well-known to increase the vaccination rate among Hajj pilgrims [79]. A short educational training program for Egyptian military nurses on selected mass gathering infectious diseases was found to improve their knowledge about vaccinations, particularly accurate knowledge about influenza vaccines and some knowledge about antiviral drugs [80].

Increasing AMR is widely acknowledged as a threat to global health, security, and development due to higher healthcare costs and decreases in labour, productivity, household incomes, and national income [81]. AMR is estimated in the European Union to cause 25,000 additional deaths and EUR 1500 million worth of extra health care costs and loss of productivity annually [82]. In the US, an excess of two million infections are caused by bacteria resistant to first-line antibiotics, with an estimated USD 20 billion of excess healthcare cost per annum [81]. Accurate estimates from low- to middle-income countries are lacking, but the burden of AMR globally is, and will be, disproportionately felt by these countries [83]. In 2015, WHO developed a Global Action Plan on AMR to ensure the ongoing ability to successfully treat and prevent infectious diseases; it was anticipated that rigorous surveillance and data collection on AMR would help to provide quality data for tackling the issue [8]. The findings from this systematic review highlight the importance that mass gatherings may play in the rising rates of AMR globally. Mass gatherings pose unique risk factors that may contribute to the spread of AMR, including settings that promote overcrowding, the presence of susceptible and advancing age populations, antibiotics that are easily accessible over the counter, and pathogens that are diverse due to the international origins of pilgrims [84]. Specifically, attendance at mass gatherings appears to be associated with the acquisition of AMR, particularly amongst *E. coli* and *S. pneumoniae* carriage isolates.

This study is limited in the breadth of pathogens with published data that could be included, with only two articles describing AMR in tuberculosis and one in influenza that met the inclusion criteria [6,46,54]. Additionally, the majority of included studies were set during religious mass gatherings, and care must be taken with the generalisation of results to all mass gatherings. Only a handful of longitudinal studies that reported rates of AMR pre- and post-attendance at mass gatherings were identified with generally small numbers of participants and isolates. With significant heterogeneity in study methodologies, a meta-analysis of the results was unable to be performed. This review is also limited in its inclusion of only manuscripts published in English, and manuscripts that dealt with forcibly displaced people dwelling in communities were excluded. This linguistic bias may have resulted in oversight of important data published in another language. Finally, few studies involved a non-exposed cohort, which compromises the quality of evidence; future studies should consider involving a comparable non-exposed cohort.

## 5. Conclusions

Within the limitations of the published studies and available data, this systematic review has identified a high prevalence of AMR in specific bacterial pathogens, namely *E. coli* and *S. pneumoniae*, isolated from individuals attending mass gatherings, primarily Hajj. In addition, there appears to be the acquisition of bacteria harbouring AMR profiles/genes in participants after they have attended mass gathering events. The findings indicate a critical need for enhanced surveillance and stringent antimicrobial stewardship policies tailored to these events. Policy implications also include the development of targeted guidelines for antimicrobial use before, during, and after these events and investment in point-of-care diagnostic tools to quickly detect resistant strains. Preventive strategies, such as pre-event health screening and post-event monitoring, alongside public health education campaigns focusing on infection control measures and vaccination, could mitigate the spread of AMR. However, this systematic review also highlights the limitations in the data that are currently available from which to make accurate assessments regarding the association between attendance at mass gatherings and the acquisition and spread of AMR. Thus, greater focus on this topic is required with well-conducted longitudinal studies set in a variety of mass gathering settings to answer these questions more comprehensively.

## Figures and Tables

**Table 1:** All included studies reporting antimicrobial resistance in any pathogen associated with a mass gathering event.

Author (Publication Year)	Study Type	Mass Gathering	Country (Study Setting)	Clinical Setting	Syndrome	Pathogens of Interest	NOS Score
Abd El Ghany et al. (2017) [17]	Cross-sectional	Hajj	Saudi Arabia	Emergency/ Outpatient clinic	Enteric infection	*Campylobacter jejuni*	4
						*E. coli*	
						*Salmonella* spp.	
						*Shingella* spp.	
						*Vibrio cholerae*	
						*Yersinia enterocolitica*	
Alyamani et al. (2017) [18]	Cross-sectional	Hajj	Saudi Arabia	Emergency/ outpatient clinic	Urinary tract infection	*E. coli*	2
Alzeer et al. (1998) [19]	Cross-sectional	Hajj	Saudi Arabia	Hospital	Pneumonia	*E. coli/Serratia* spp.	4
						*K. pneumoniae*	
						*Legionella/Mycoplasma pneumoniae*	
						*Mycobacterium tuberculosis*	
						*S. pneumoniae*	
Ashgar et al. (2013) [20]	Cohort	Hajj and Umrah	Saudi Arabia	Airport	Nasopharyngeal carriage	*Neisseria meningitidis*	6
Baharoon et al. (2009) [21]	Cross-sectional	Hajj	Saudi Arabia	ICU	Severe sepsis	Anaerobes	4
						*Candida* spp.	
						*Enterobacteriaceae* (*K. pneumoniae*, *E. coli*, *Enterobacter* spp., *Proteus* spp., *Citrobacter* spp.)	
						*M. tuberculosis*	
						Other Gram-negative bacteria (*Haemophilus influenzae*, *Moraxella catarrhalis*)	
						Other Gram-positive bacteria (*S. pneumoniae*, *Enterococcus* spp., Coagulase-negative staphylococci)	
						*Pseudomonas* spp.	
						*S. aureus*	
Zumla and Memish [22] †	Case series	Hajj or Umrah	England	Hospital	Meningococcal conjunctivitis	*N. meningitidis*	N/A
Bukhari et al. (2008) [23]	Case report	Hajj	Saudi Arabia	ICU	Pneumonia	*Ewingella americana*	N/A
Crampin et al. (1999) [24]	Outbreak investigation	Glastonbury music festival	England	Community	Enteric infection	*E. coli*	5
Fatani et al. (2002) [25]	Cross-sectional	Hajj	Saudi Arabia	Outpatient clinic	Pyoderma	Gram-negative bacilli (*Pseudomonas aeruginosa*, *E. coli*, *Proteus* and *Klebsiella* spp.)	4
						*S. aureus*	
						*Streptococcus pyogenes*	
Francois Watkins et al. (2020) [26]	Case series	Arbaeen	United States of America	NR	Enteric fever	*Salmonella enterica* subsp. *enterica* serovar Typhi	N/A
Ganaie et al. (2018) [27]	Cohort	Hajj	India	NR	Naso-/oro-pharyngeal carriage	*S. pneumoniae*	6
Godbole et al. (2019) [28]	Case report	Arba’een	Iraq	Community	Enteric fever	*S.* Typhi	N/A
Gorla et al. (2012) [29]	Outbreak investigation	Party	Brazil	Hospital	Meningococcal disease	*N. meningitidis*	3
Haseeb et al. (2016) [30]	Retrospective review of hospital records	Hajj and Umrah	Saudi Arabia	Hospital	Community-acquired infections	*Acinetobacter baumannii*	3
						*Enterobacter cloacae*	
						*E. coli*	
						*K. pneumoniae*	
						*Proteus mirabilis*	
						*P. aeruginosa*	
						*Salmonella* spp.	
						*S. aureus*	
						*Streptococcus* spp.	
Johargy et al. (2011) [31]	Cohort	Hajj and Umrah	Saudi Arabia	Community	Nasopharyngeal carriage	*S. aureus*	4
Leangapichart et al. (2016) [32]	Cohort	Hajj	Saudi Arabia	Community	Gastrointestinal carriage	*E. coli*	5
						*K. pneumoniae*	
Leangapichart et al. (2016) [33]	Cohort	Hajj	Saudi Arabia	Community	Gastrointestinal carriage	*A. baumannii*	5
						*E. coli*	
Leangapichart et al. (2016) [34]	Cohort	Hajj	Saudi Arabia	Community	Gastrointestinal carriage	*E. coli*	5
						*K. pneumoniae*	
Leangapichart et al. (2017) [35]	Cohort	Hajj	Saudi Arabia/France	Community	Gastrointestinal carriage	NR	5
Marglani et al. (2016) [36]	Cross-sectional	Hajj	Saudi Arabia	Emergency/ outpatient clinic	Acute rhinosinusitis	*Citrobacter* spp.	4
						*Enterobacter aerogenes*	
						*E. coli*	
						*Klebsiella oxytoca*	
						*K. pneumoniae*	
						*Proteus vulgaris*	
						*S. aureus*	
Memish et al. (2015) [37]	Cross-sectional	Hajj	Saudi Arabia	NR	Nasopharyngeal carriage	*S. pneumoniae*	5
Memish et al. (2006) [38]	Cross-sectional	Hajj	Saudi Arabia	Outpatient clinic	Nasal/skin carriage	*S. aureus*	4
Memish et al. (2016) [39]	Cohort	Hajj	Saudi Arabia	Community	Nasopharyngeal carriage	*S. pneumoniae*	6
Olaitan et al. (2015) [40]	Cohort	Hajj	France	Community	Gastrointestinal carriage	*Salmonella enterica* subsp. *enterica* serovar *Newport*	5
Osman et al. (2018) [41]	Cross-sectional	El-Tahrir Square protest	Egypt	Outpatient clinic	Urinary tract infection	*E. coli*	3
Shirah et al. (2017) [42]	Retrospective review of hospital records	Hajj	Saudi Arabia	Hospital	Pneumonia	*S. aureus*	3
Ng and Taha (1994) [43]	Case series	Hajj or Umrah	Malaysia	Hospital	Enteric infection	*V. cholerae*	N/A
Wharton et al. (1990) [44]	Outbreak investigation	Rainbow family gathering	United States of America	Community	Enteric infection	*Shigella sonnei*	6
Yousuf and Nadeem (2000) [45]	Prospective cohort	Hajj and Umrah	Saudi Arabia	Hospital	Meningococcal disease	*N. meningitidis*	4
Yezli et al. (2017) [46]	Cross-sectional	Hajj	Saudi Arabia	Community	Tuberculosis	*M. tuberculosis*	4
Ziyaeyan et al. (2012) [16]	Cross-sectional	Hajj	Iran	Community	Upper respiratory tract infection	Influenza A(H1N1)pdm09	4
Al-Zahrani et al. (2019) [47]	Cross-sectional	Hajj and Umrah	Saudi Arabia	Hospital	SSTI/bacteraemia/others	*S. aureus*	3
Bokhary et al. (2022) [48]	Cross-sectional	Hajj	Saudi Arabia	Primary health care centre	Upper respiratory tract infection	*H. influenzae*, *S. aureus*, *S. pneumoniae*, *M. catarrhalis*	5
Yezli et al. (2022) [49]	Prospective cross-sectional	Umrah	Saudi Arabia	Pilgrims’ places of residence	Meningococcal carriage	*N. meningitidis*	6
Hoang et al. (2021) [50]	Prospective cohort study	Hajj	France and KSA	Community	Acquisition of multidrug-resistant bacteria	MRSA, ESBL-E, CRAB, *E. aerogenes*, *K. pneumoniae*, *E. coli*, *E cloacae*, *Citrobacter koseri*, and *S. aureus*	6
Leangapichart et al. (2021) [51]	Comparative genomics study	Hajj	France and KSA	Community	Diarrhea at Hajj	*Shewanella xiamenensis*	3
Booq et al. (2022) [52]	Cross-sectional study	Umrah	KSA	Hospital	Isolation of antibiotic-resistant *K. pneumoniae* during Umrah	*K. pneumoniae*	3
Harimurti et al. (2021) [53]	Prospective longitudinal study	Hajj	KSA	Community	Pneumococcal carriage	*S. pneumoniae*	6
Yezli et al. (2023) [54]	Prospective cross-sectional study	Hajj	KSA	Hospital and non-hospital settings	Active pulmonary tuberculosis	*M. tuberculosis*	7
Baharin et al. (2021) [55]	Cross-sectional study	Hajj	Malaysia	Airport	URTI	Influenza A	5
						MERS-CoV	
						Mycobacterium bovis	
						*S. pneumoniae*	
						*K. pneumoniae*	
Yezli et al. (2023) [56]	Longitudinal Cohort	Hajj	Saudi Arabia	Community	Meningococcal carriage	*N. meningitidis*	7
Ouaddane et al. (2024) [57]	Cohort	Grand Magal de Touba	Senegal	Community	*S. aureus* carriage	*S. aureus*	5
Ouaddane et al. (2024) [58]	Cohort	Grand Magal de Touba	Senegal	Community	Gastrointestinal symptoms	Gastrointestinal bacteria	6
Dao et al. (2024) [59]	Prospective cohort	Hajj	France and KSA	Private specialist travel agency	MRSA carriage	*S. aureus*	6

† This publication is based on a source report from Public Health England titled ‘Ciprofloxacin resistant cases of non-groupable meningococcal infection connected to recent travel to Mecca. 2019.’ The weblink to the source report has since expired and is no longer available online. CRAB = Carbapenem-resistant *Acinetobacter baumannii*; ICU = intensive care unit; MERS-CoV = Middle East respiratory syndrome coronavirus; N/A = not applicable; NR = not reported; NOS = Newcastle–Ottawa Scale; spp. = species; SSTI = skin and soft tissue infection; URTI = upper respiratory tract infection; KSA = Kingdom of Saudi Arabia; MRSA = Methicillin resistant *S. aureus*.

**Table 2:** Included studies with individual laboratory and statistical methods and summary findings by mass gathering category.

Author	Laboratory Methods	Comparisons Made in Each Study	Statistical Methods Applied in Data Analysis	Key Pathogens Identified in This Group of Studies	Key AMR Findings of This Group of Studies
**Hajj and Umrah Studies**				*E. coli*, *K. pneumoniae*, *S. pneumoniae*, MRSA, *M. tuberculosis*, *N. meningitidis.*	Increased prevalence of ESBL-producing *E. coli* and *K. pneumoniae*. Post-Hajj rise in nasopharyngeal carriage of AMR pathogens. No rifampicin resistance detected in TB cases.
Ganaie et al. (2018) [27]	*S. pneumoniae* isolates were identified using colony morphology, Gram staining, optochin susceptibility, and bile solubility tests. Serotyping of pneumococcal isolates was performed by Quellung reaction. Antimicrobial susceptibility testing of the isolates was performed by broth microdilution. DNA was extracted and used for quantitative multiplex real-time PCR.	The study compared the *S. pneumoniae* carriage before and after Hajj pilgrimage. It also compared the prevalence of *S. pneumoniae* detected by culture and quantitative multiplex real-time PCR. Additionally, the study compared the number of pilgrims carrying multiple serotypes before and after pilgrimage.	Paired *t*-tests were performed to analyse pre- and post-Hajj cohorts and chi-squared tests were performed on categorical data.		
Al-Zahrani et al. (2019) [47]	*S. aureus* isolates were identified using the Vitek 2 system. SmaI-multiplex PCR typing (SMT) was carried out for all isolates. PCR and sequencing were used to perform multi-locus sequence typing (MLST) and SCCmec typing.	SmaI-multiplex PCR typing (SMT) and Multi-Locus Sequence Typing (MLST) were compared to determine utility of SMT as an alternative to MLST for monitoring the impact of mass migration on the clonality of *S. aureus*.	Descriptive statistics and cluster analysis.		
Abd El Ghany et al. (2017) [17]	Enzyme immunoassays were used to detect viral and parasitic pathogens. Total DNA was isolated and purified from faecal samples and used for molecular characterisation of bacterial species by multiplex PCR. Viral RNA was extracted from antigenically positive samples and used for molecular characterisation of viral agents by RT-PCR. The samples were screened for the detection of β-lactamase genes by PCR.	The study compared the frequency of clinical symptoms of patients across three Hajj seasons. The study also compared the distribution of infectious agents across the three Hajj seasons. Additionally, the study compared the percentage of samples with identified etiologic agents and bacterial agents in patients with severe and mild symptoms.	Pearson χ^2^ test was used to evaluate differences between sets of categorical data.		
Fatani et al. (2002) [25]	Skin swabs were collected and cultured on sheep blood agar and MacConkey agar. Organisms were identified by standard microbiological methods. Susceptibility testing was performed using the Kirby–Bauer disc diffusion method.	Comparison of primary and secondary pyoderma cases: types of organisms isolated, demographic details (age, gender, nationality), antibiotic resistance patterns, and incidence rates of different pyoderma subtypes.	Not explicitly reported; descriptive analyses and percentage-based comparisons were used.		
Dao et al. (2024) [59]	Nasopharyngeal swab collection pre- and post-Hajj; DNA extraction for real-time PCR for *S. aureus* using the *nucA* gene and real-time PCR for MRSA using *mecA* and *mecC* genes.	Prevalence of *S. aureus* pre- and post-Hajj; Prevalence of MRSA pre- and post-Hajj; Acquisition rates of *S. aureus* and MRSA during the Hajj; Comparison of clinical features between pilgrims colonised with MRSA versus those negative with MRSA.	Not specified.		
Leangapichart et al. (2016) [32]	*E. coli* and *K. pneumoniae* isolates were tested against six antibiotics using the disk diffusion method. Total DNA was extracted, and ESBL-encoding genes were detected by PCR and sequencing. MLST was performed, and a phylogenetic tree was constructed.	The study compared the proportions of pilgrims harbouring antibiotic-resistant *E. coli* and *K. pneumoniae* before and after the Hajj pilgrimage. The study also compared the number of pilgrims harbouring ESBL genes before and after the pilgrimage.	McNemar’s or Fisher’s exact test was used to calculate the change in the number of pilgrims harbouring antibiotic-resistant bacteria and ESBL genes.		
Leangapichart et al. (2016) [33]	Pharyngeal and rectal swabs were collected and cultured, and bacterial species were identified. Antibiotic susceptibility testing was performed. Real-time PCR was used to identify *A. baumannii* and carbapenemase-producing bacteria. MLST was performed on nonduplicate *A. baumannii* isolates.	The study compared the bacterial diversity in pharyngeal swab samples taken from pilgrims before and after the Hajj pilgrimage. The study also compared the sequence types of *A. baumannii* isolates from pharyngeal and rectal swab samples.	A chi-squared test was used to compare bacterial species diversity between pre- and post-Hajj samples.		
Marglani et al. (2016) [36]	Samples were collected from the middle meatus, stained with Gram stain, and cultured. Identification and antimicrobial susceptibility testing were performed using the MicroScan Walk Away System. Susceptibility testing for *S. pneumoniae* and *H. influenzae* was performed using the disk diffusion method.	The study compared the severity of symptoms associated with different bacterial species. The study also compared the total Modified Arabic Sinonasal Outcome Test (MA-SNOT) scores between bacterial and non-bacterial groups.	Not specified, but reported descriptive statistics.		
Leangapichart et al. (2016) [34]	Real-time PCR: Screening for *mcr-1* gene in rectal swabs. Culture: Isolation of colistin-resistant strains on Cepacia agar. MALDI-TOF: Bacterial species identification. Antibiotic susceptibility testing: Using EUCAST guidelines. E-test: colistin MIC determination. PCR and sequencing: detection of *mcr-1* and ESBL genes (*bla_CTX-M_*, *bla_TEM_*, *bla_SHV_*). Multilocus sequence typing (MLST) of *E. coli* and *K. pneumoniae* isolates.	Compared the prevalence of *mcr-1*-positive isolates in rectal swab samples before and after the pilgrimage.	Not specified, but reported descriptive statistics.		
Haseeb et al. (2016) [30]	Retrospective audit of patient records admitted to two hospitals in Makkah from January to June 2015. Infections categorised as community-acquired based on positive cultures within 72 h of admission. Antimicrobial susceptibility testing performed according to Clinical and Laboratory Standards Institute standards (25th informational supplement, M100-S25).	Compared the resistance patterns of different bacterial pathogens (Gram-positive and Gram-negative) to various antibiotics. Did not compare resistance patterns before and after the pilgrimage.	Not specified, but reported descriptive statistics and comparative statistics.		
Memish et al. (2016) [39]	Nasopharyngeal swabs, microbiological culture, serotyping, and MIC determination.	Pre- vs. post-Hajj carriage rates, serotype coverage by vaccines.	Statistical significance tests.		
Olaitan et al. (2015) [40]	Rectal swabs, quantitative PCR, culture, MALDI-TOF, MIC testing, whole-genome sequencing.	Pre- vs. post-Hajj acquisition of multidrug-resistant *Salmonella.*	Not reported.		
Memish et al. (2015) [37]	Nasopharyngeal swabs, microbiological identification, serotyping, and MLST.	Beginning-Hajj vs. End-Hajj carriage rates, serotypes, and antibiotic resistance.	Bivariate and multivariate analyses, prevalence ratios, adjusted odds ratios.		
Shirah et al. (2017) [42]	Retrospective analysis of pneumonia cases.	Incidence, risk factors, microbial patterns of pneumonia; differences in pathogens between Hajj and general settings.	Chi-squared test; descriptive statistics.		
Yousuf and Nadeem (2000) [45]	CSF and blood cultures; Commercial latex agglutination kit used for serotyping of the meningococci; antimicrobial sensitivity testing.	Mortality across serogroups; clinical and demographic variations (e.g., age, nationality). Susceptibility of isolates to antibiotics.	Descriptive statistics.		
Ashgar et al. (2013) [20]	Nasopharyngeal swabs, cultured on chocolate agar and Thayer Martin agar, Gram stain, VITEK 2 system.	Comparison of carriage rates before and after Umrah and Hajj among different nationalities.	Chi-squared tests.		
Ng and Taha (1994) [43]	Rectal swabs, disc-diffusion method for antibiotic susceptibility.	Comparison of antibiotic resistance patterns among the three reported cases.	Not specified but reported descriptive statistics and comparative statistics.		
Alzeer et al. (1998) [19]	Sputum culture, broncho-alveolar lavage, microscopy, serological assays for *Mycoplasma* and *Legionella*.	Comparison of causative organisms in pneumonia cases among pilgrims, specifically looking at tuberculosis vs. others.	Student’s *t*-test to detect significant differences between groups.		
Bukhari et al. (2008) [23]	Blood culture.	A case report of *E. americana* infection in an immunocompromised patient with pneumonia.	Not reported.		
Baharoon et al. 2009 [21]	Blood culture and chest X-ray.	Comparison of incidence and outcomes of severe sepsis and septic shock among pilgrims.	Not reported.		
Memish et al. (2006) [38]	Standard microbiological techniques were used to screen for the presence of MRSA. *S. aureus* with cefoxitin zones of ≤19 mm reported as oxacillin-resistant.	Compared the prevalence of *S. aureus* found in the nares, axillae, and both. Compared prevalence of MRSA in the study population to prevalence found in other community-based studies. Compared gender, age, nationality, residence, hospitalisation, and *S. aureus* culture results.	Chi-squared test.		
Johargy et al. (2011) [31]	Nasal swabs were cultured on mannitol salt agar to grow *S. aureus.*	Compared the prevalence of *S. aureus* in Umrah visitors before and after performing Umrah. Compared the prevalence of *S. aureus* in pilgrims before and after performing Hajj. Compared prevalence of *S. aureus* in different nationalities of Umrah visitors.	Chi-squared test.		
Leangapichart et al. (2017) [35]	CTX-M genes in rectal samples were identified by PCR and confirmed by sequencing.	Compared prevalence of CTX-M genes (*bla*_CTX-M_) in pilgrims pre- and post-Hajj.	Pearson χ^2^ test and Fisher’s exact test, Student’s *t*-test, and logistic regression.		
Yezli et al. (2023) [56]	Oropharyngeal swabs cultured on selective agar; whole-genome sequencing; antibiotic susceptibility testing; serogrouping.	Pre-Hajj vs. post-Hajj carriage rates; antimicrobial resistance profiles between isolates.	Descriptive statistics.		
Alyamani et al. (2017) [18]	Phenotypic and genotypic testing (Vitek system, PCR); MIC assays; MLST.	Distribution of resistance genes among isolates; resistance profiles across antibiotic classes.	Not explicitly mentioned, but methods include frequency and percentage calculations for phenotypic resistance.		
Ziyaeyan et al. 2012 [16]	Real-time RT-PCR for RNA detection; oseltamivir resistance testing using a genotyping kit.	Prevalence of A(H1N1)pdm09 among pilgrims; viral load in relation to fever and symptoms.	Fisher’s exact test.		
Yezli et al. (2017) [46]	Xpert MTB/RIF assay for *M. tuberculosis* (TB) and rifampicin resistance detection.	Prevalence of undiagnosed tuberculosis among pilgrims by age, gender, education level, and comorbidities.	Chi-squared test, Fisher’s exact test, logistic regression analysis (Firth method).		
Yezli et al. (2022) [49]	Oropharyngeal swabs plated on Neisseria-selective agar, Gram staining, and biochemical tests for identification, serogrouping using antigenic assays, and antibiotic susceptibility testing using E-tests.	Prevalence of *N. meningitidis* carriage among pilgrims; distribution of serogroups; antibiotic resistance profiles.	Not reported.		
Leangapichart et al. (2021) [51]	Real-time PCR; conventional PCR and sequencing; culture on MacConkey agar with ertapenem; MALDI-TOF; whole-genome sequencing using the Illumina MiSeq platform.	Comparisons of the draft genome sequences of two *S. xiamenensis* isolates with other closely related genomes.	Not specifically re-ported, but conducted comparative genomic analysis between environmental and human strains to describe the genomic diversity.		
Harimurti et al. (2021) [53]	Nasopharyngeal swab collection; *S. pneumoniae* identification using conventional and molecular approaches; antibiotic susceptibility determination using a disk diffusion method; serotyping by sequential multiplex PCR (smPCR) targeting the *wzy* gene; confirmation of non-typeable isolates by real-time PCR targeting *lytA.*	Comparison of the prevalence of *S. pneumoniae* carriage rates before and after Hajj; comparison of the five most prevalent serotypes before and after Hajj; comparison of the percentage of isolates susceptible to co-trimoxazole before and after Hajj.	McNemar test.		
Hoang et al. (2021) [50]	Nasopharyngeal and rectal swabs; culture on specific media (MRSA agar, MacConkey agar, SMART agar, VRE agar); identification using MALDI-TOF; antibiotic susceptibility testing using the disk diffusion method; DNA extraction using QIAamp DNA Mini Kit; detection of antibiotic resistance-encoding genes using quantitative PCR.	Proportions of pilgrims acquiring MDR bacteria during the Hajj; prevalence of MDR bacteria pre- and post-Hajj; distinction between acquisition and persistent carriage of MDR bacteria based on species, anatomical sites, antibiotic susceptibility, and genetic characteristics; prevalence of colistin resistance genes pre- and post-Hajj.	Pearson χ^2^ test; Fisher’s exact test; McNemar’s test; univariable analysis; multivariable analysis; logistic regression.		
Booq et al. (2022) [52]	Bacterial identification using MicroScan and VITEK2; MIC determination using the microdilution method; biofilm formation assay using crystal violet staining and quantification; detection of plasmids using PCR-based replicon typing (PBRT 2.0 kit); detection of carbapenem resistance genes and virulence genes using conventional PCR.	Antibiotic resistance profiles of *K. pneumoniae* isolates; biofilm formation capabilities of *K. pneumoniae* isolates; prevalence of plasmid replicons among isolates; presence and co-occurrence of carbapenem resistance genes; distribution of virulence genes.	Not reported, but authors mention the use of mean and standard deviation to report the results of the biofilm formation assay.		
Yezli et al. (2023) [54]	Sputum sample collection; *M. tuberculosis* detection using Xpert MTB/RIF assay.	Comparison of tuberculosis prevalence between hospitalised and non-hospitalised pilgrims; association of various factors (e.g., age, underlying conditions, cough in household, and cough length) with tuberculosis in both hospitalised and non-hospitalised pilgrims.	Descriptive statistics; bivariate analysis; multivariate logistic regression using the Firth method.		
Baharin et al. (2021) [55]	Throat swab collection; nucleic acid extraction; PCR assays for pathogen detection (influenza A, MERS-CoV, *M. bovis*, *S. pneumoniae*, and *K. pneumoniae*); PCR assays for virulence and antibiotic resistance genes.	Prevalence of *S. pneumoniae* and *K. pneumoniae*; prevalence of virulence genes in *S. pneumoniae* and *K. pneumoniae* isolates; prevalence of antibiotic resistance genes in *S. pneumoniae* and *K. pneumoniae* isolates; distribution of respiratory tract infection symptoms among pilgrims.	Not specified.		
Bokhary et al. (2022) [48]	Oropharyngeal swab collection; culturing on blood agar, chocolate agar, and MacConkey agar; bacterial identification using the VITEK 2 COMPACT system; antimicrobial susceptibility testing using VITEK 2 COMPACT systems and Kirby–Bauer disc diffusion method.	Comparison of bacterial infection rates among different demographic groups; comparison of antibiotic prescription rates and appropriateness; assessment of the predictive value of clinical findings for bacterial infection.	Sensitivity, specificity, positive predictive value, negative predictive value, odds ratio, and confidence intervals.		
**Arbaeen studies**				*Salmonella* Typhi.	Reports of ceftriaxone-resistant *Salmonella* Typhi with ESBL genes (*bla_CTX-M-15_*).
Francois Watkins et al. (2020) [26]	Antimicrobial susceptibility testing; whole-genome sequencing.	Comparison of *Salmonella* Typhi isolates from travellers to Pakistan and Iraq; comparison of antibiotic resistance patterns between *S.* Typhi isolates; comparison of patient characteristics (age, sex, travel history, vaccination status, hospitalisation) between those with XDR *S.* Typhi and those with non-XDR *S.* Typhi	Not specified.		
Godbole et al. (2019) [28]	Blood culture; whole-genome sequencing.	Phylogenetic analysis to compare the Iraqi *Salmonella* Typhi strain to strains from a Public Health England database and other previously described strains.	Not specified.		
**Grand Magal de Touba studies**				*E. coli*, *K. pneumoniae*, *S. pneumoniae*, MRSA.	The prevalence of gastrointestinal bacteria was high both pre-Magal (48.5%) and post-Magal, and no increase in post-Magal MRSA acquisition was noted.
Ouaddane et al. (2024) [57]	Nasopharyngeal sampling; DNA extraction using the EZ1 Advanced XL with the DNA Tissue Kit; PCR screening for *S. aureus* using the *nucA* gene; real-time PCR screening for *mecA* and *mecC* genes.	Prevalence of *S. aureus* carriage pre- and post-Grand Magal de Touba; prevalence of *mecA* and *mecC* genes pre- and post-Grand Magal de Touba; acquisition rates of *S. aureus*, *mecA*, and *mecC* during the Grand Magal de Touba.	Not specified.		
Ouaddane et al. (2024) [58]	Rectal swab collection pre- and post-Grand Magal de Touba (GMT); DNA extraction; quantitative PCR for gastrointestinal bacteria and resistance genes. Culture on specific media for VRE, MRSA, ESBL producing, and carbapenemase-positive bacteria; bacterial identification using MALDI-TOF; antibiotic susceptibility testing using the Kirby–Bauer disk diffusion method.	Prevalence of gastrointestinal bacteria pre- and post-GMT; prevalence of resistance genes pre- and post-GMT; association between acquisition of resistance genes and demographic, medical, preventive, therapeutic, and clinical factors.	Pearson χ^2^ test; Fisher’s exact test; univariate analysis; multivariate analysis using logistical regression.		
**Other mass gatherings (entertainment and political protest)**				*E. coli*, *Shigella* spp., *N. Meningitidis*.	MDR *E. coli* among attendees of El-Tahrir Square Protest and at a music festival, and drug-resistant meningococcal and *Shigella* isolates at parties.
Osman et al. (2018) [41]	PCR for virulence and resistance genes; phylogenetic classification; antimicrobial susceptibility testing; biofilm assay; serotyping using O-antisera; phenotypic assays for motility and haemolysis.	Resistance profiles of *E. coli* strains; biofilm formation patterns across phylogenetic groups; prevalence of specific virulence genes.	Not explicitly mentioned.		
Crampin et al. 1999 [24]	Faecal specimens were plated on sorbitol MacConkey agar; putative *E. coli* O157 were identified by agglutination with antiserum to the O157 antigen; isolates underwent serotyping, phage typing, and Vero cytotoxin (VT) gene probing; VT2 gene subtype was determined by PCR; DNA of the VTEC O157 isolates was digested with restriction enzyme Xba I and compared by pulsed-field gel electrophoresis; serum samples were screened for the presence of antibodies to the O157 antigen; rectal swabs from cows were collected and tested.	The study compared *E. coli* O157 strains from seven people and from a cow belonging to a herd that had previously grazed at the festival site. Drug resistance and DNA-based tests were also used to compare strains.	Not specified, but reported descriptive statistics, prevalence estimation and comparative analysis.		
Gorla et al. (2012) [29]	Meningococcal strains were serogrouped using conventional microbiologic methods; DNA was prepared in agarose plugs and digested with the restriction enzyme Nhe I; serotyping and serosubtyping were performed by dot blotting using whole-cell suspensions and murine Mabs; MLST was performed and antimicrobial susceptibility testing was performed using the broth microdilution procedure.	The study compared restriction profiles from *N. meningitidis* isolates to determine relatedness.	Not specified.		
Wharton et al. (1999) [44]	Stool cultures for pathogens; antimicrobial susceptibility testing; plasmid profiling; colicin typing.	Attack rates among genders, age groups, water usage, and communal kitchen exposure; resistance profiles.	Univariate (relative risks) and multivariate logistic regression.		

AMR = antimicrobial resistance; MRSA = methicillin-resistant S. aureus; PCR = polymerase chain reaction; EUCAST = European Committee on Antimicrobial Susceptibility Testing; ESBL = extended-spectrum beta-lactamase; DNA = deoxyribonucleic acid; RNA = ribonucleic acid; MALDI-TOF = matrix-assisted laser desorption ionisation–time of flight; MIC = minimum inhibitory concentration; MLST = multi-locus sequence typing; CSF = cerebrospinal fluid; MDR = multidrug-resistant; MERS-CoV = Middle East respiratory syndrome coronavirus; XDR = extensively drug-resistant; VRE = vancomycin-resistant *Enterococcus*.

**Table 3:** Longitudinal cohort studies reporting antimicrobial resistance in *E. coli* carriage isolates identified among pilgrims attending a mass gathering *.

Author (Publication Year)	Phenotypic Resistance			Genotypic Resistance		
	Antibiotic	Pre-Mass Gathering Number (%)	Post-Mass Gathering Number (%)	Resistance Genes	Pre-Mass Gathering Number (%)	Post-Mass gathering Number (%)
Leangapichart et al. (2016) [32]	Colistin	3/129 (2.3)	2/129 (1.6)	ESBL genes (CTX-M, TEM, or SHV)	5/23 (21.7)	18/23 (78.3)
	Ceftriaxone †	5/129 (3.9)	18/129 (14.0)			
	Ticarcillin-clavulanic acid †	16/129 (12.4)	29/129 (22.5)			
	Gentamicin	2/129 (1.6)	9/129 (7.0)			
	Piperacillin-tazobactam	0	0			
	Imipenem	0	0			
	Any †	18/129 (14.0)	36/129 (28.0)			
Ouaddane et al. (2024) [58]	Amoxycillin-clavulanate	18/27 (66.7)	23/38 (60.5)	ARG CTX-MA CTX-MB SHV TEM OXA 23	260/295 (88.1) 68/295 (23.0) 30/295 (10.1) 154/295 (52.0) 259/295 (87.5) 10/295 (3.9)	230/291 (79.0) 82/291 (28.2) 13/291 (4.5) 124/291 (42.6) 218/291 (3.9) 1/291 (0.3)
	Amoxicillin	27/27 (100)	35/38 (92.1)			
	Amikacin	0/27 (0)	2/38 (5.3)			
	Ciprofloxacin	10/27 (37)	9/38 (23.7)			
	Ceftriaxone	27/27 (100)	31/37 (83.8)			
	Doxycycline	4/27 (14.8)	15/38 (39.5)			
	Ertapenem	0/27 (0)	1/37 (2.7)			
	Cefepime	11/27 (40.7)	13/37 (35.1)			
	Fosfomycin	2/27 (7.4)	0/38 (0)			
	Gentamicin	3/27 (11.1)	4/38 (10.5)			
	Imipenem	0/27 (0)	1/37 (2.7)			
	Cotrimoxazole	17/27 (63)	26/38 (68.4)			
	Piperacillin + Tazobactam	1/27 (3.7)	4/38 (10.5)			
Leangapichart et al. (2016) [33]	1/2 (50%) resistant to cefoxitin, ceftriaxone, cefotaxime, amoxycillin-clavulanic acid, ticarcillin-clavulanic acid, amoxycillin, tobramycin, gentamicin, amikacin, rifampicin, and trimethoprim-sulfamethoxazole.			blaNDM-5, blaCTX-M-15, blaTEM-1, aadA2	0/2 (0)	1/2 (50.0)
	1/2 (50%) resistant to multiple antibiotics except colistin and imipenem			*blaNDM-5*, *blaTEM-1*, *aadA2*	0/2 (0)	1/2 (50.0)
Leangapichart et al. (2016) [34]	Amoxycillin		10/10 (100)	Colistin resistance gene † (*mcr-1*)	2013 Hajj:	2013 Hajj:
	Amoxycillin-clavulanate		9/10 (90)		2/129 (1.6)	11/129 (8.5)
	Trimethoprim-sulfamethoxazole		7/10 (70)		2014 Hajj:	2014 Hajj:
	Ceftriaxone		4/10 (40)		1/92 (1.0)	9/90 (9.2)
	Aztreonam		2/10 (20)			
	Fosfomycin		2/10 (20)			
	Cefepime		1/10 (10)			
	Gentamicin		4/10 (40)	*bla*_TEM-1_	0	8/10 (80)
	Nalidixic acid		3/10 (30)	*bla*_SHV-1_	0	1/10 (10)
	Ciprofloxacin		1/10 (10)	*bla*_CTX-M-15_, *bla*_TEM-1_	0	1/10 (10)
Hoang et al. (2021) [50]	Penicillin		‡ (100)	TEM CTX-M-A CTX-M-A & TEM CTX-M-A & SHV TEM & SHV	1/268 (0.4) 9/268 (3.4) 3/268 (1.1) 0/268 (0) 1/268 (0.4)	2/268 (0.7) 3/268 (1.1) 21/268 (7.8) 1/268 (0.4) 1/268 (0.4)
	Ampicillin--clavulanate		‡ (57.8)			
	Cefepime		‡ (57.3)			
	Ceftriaxone		‡ (95.5)			

* All studies involved Hajj pilgrimage except one [58] which involved Grand Magal de Touba. † Statistically significant difference; ‡ numerator and denominator for this percentage are not available.

**Table 4:** Cross-sectional and other descriptive reporting antimicrobial resistance in *E. coli* isolates identified in the setting of mass gatherings.

First Author (Publication Year)	Phenotypic Resistance		Genotypic Resistance	
	Antibiotic	Number (%)	Resistance Gene	Number (%)
Alyamani et al. (2017) [18]	Ampicillin	56/58 (96.6)	*aac6* *aac61b* *aadA4* *strB* *aadA1* *aadA2* *aadB* *ant2* *aphA* *strA* *bla*_CTX-M-1_, *bla*_CTX-M-15_, *bla*_OXA-1_, *bla*_TEM-1_ *bla*_CTX-M-1_, *bla*_CTX-M-15_, *bla*_OXA-1_ *bla*_OXA-1_, *bla*_TEM-1_ *bla*_SHV_	26/58 (44.8) 25/58 (43.0) 24/58 (42.0) 21/58 (36.0) 9/58 (15.0) 7/58 (12.0) 2/58 (4.0) 2/58 (4.0) 7/58 (12.0) 1/58 (1.0) 8/58 (13.7) 10/58 (17.2) 2/58 (3.4) 2/58 (3.4)
	Cefoxitin	9/58 (15.3)		
	Ciprofloxacin	46/58 (79.7)		
	Cefepime	43/58 (74.6)		
	Aztreonam	52/58 (89.8)		
	Cefotaxime	44/58 (76.3)		
	Ceftazidime	47/58 (81.4)		
	Meropenem	0		
	Imipenem	0		
Crampin et al. (1999) [24]	Sulphonamides	6/8 (75.0)		
	Tetracycline	6/8 (75.0)		
Abd El Ghany et al. (2017) [17]	NR	NR	*bla*_CTX-M-15_, *bla*_NDM_	16/48 (33.3)
Haseeb et al. (2016) [30]	Amoxycillin-clavulanate	38/65 (59.0)		
	Ampicillin	66/825 (8.0)		
	Ceftazidime	20/1000 (2.0)		
	Amikacin	16/229 (7.0)		
	Ciprofloxacin	57/95 (60.0)		
	Levofloxacin	4/4 (100.0)		
Marglani et al. (2016) [36]	Ampicillin	2/8 (25.0)		
	Trimethoprim-sulfamethoxazole	7/8 (87.5)		
Osman et al. (2018) [41]	Tetracycline, nalidixic acid, ampicillin, trimethoprim, neomycin, oxytetracycline and erythromycin Colistin	29/29 (100.0)	*tet(A)* *sulI* *sulII* *dhfrI* *dhfrXIII*	7/29 (24.1) 12/29 (41.4) 14/29 (48.3) 17/29 (58.6) 12/29 (41.4)
		5/29 (17.2)		

NR = not reported.

**Table 5:** Antimicrobial resistance in *K. pneumoniae* isolates identified among pilgrims attending mass gatherings.

First Author (Publication Year)	Phenotypic Resistance			Genotypic Resistance		
	Antibiotic	Pre-Mass Gathering Number (%)	Post-Mass Gathering Number (%)	Resistance Genes	Pre-Mass Gathering Number (%)	Post-Mass Gathering Number (%)
Leangapichart et al. (2016) [32]	Ceftriaxone	0	5/129 (3.9)	*bla*_CTX-M-15_ (_+TEM_ and _SHV_) *bla*_CTX-M-14_ + *bla*_SHV-161_	0 0	4/5 (80.0) 1/5 (20.0)
	Gentamicin	0	4/129 (3.1)			
	Ticarcillin/clavulanate	19/129 (14.7)	22/129 (17.1)			
	Colistin	5/129 (3.9)	4/129 (3.1)			
	Any antibiotic	22/129 (17.1)	24/129 (18.6)			
Leangapichart et al. (2016) [34]	Amoxycillin, amoxycillin–clavulanic acid, Trimethoprim-sulfamethoxazole	0	1/1 (100.0)	*mcr1* and *bla*_TEM-1_	0	1/1 (100.0)
Booq et al. (2022) [52]	Cefoxitin	--	22/23 (95.7)	*blaVIM* *blaOXA-48* *blaNDM-1* *blaOXA-23-like*	-- -- -- --	23/23 (100.0) 20/23 (87.0) 7/23 (30.4) 1/23 (4.3)
	Cefepime		22/23 (95.7)			
	Aztreonam		22/23 (95.7)			
	Ceftazidime		22/23 (95.7)			
	Cefotaxime		23/23 (100.0)			
	Ciprofloxacin		22/23 (95.7)			
	Meropenem		21/23 (91.3)			
	Imipenem		22/23 (95.7)			
	Ampicillin		23/23 (100.0)			
Baharin et al. (2021) [55]	The study only tested for resistance genes, not for specific antibiotic resistance	--	--	*blaKPC*	--	2/4 (50.0)
				*blaOXA-48*	--	2/4 (50.0)
				*mefA gene*	--	5/13 (38.5)
				*mefA* & *pbpA*	--	5/13 (38.5)
				*pbpA*	--	2/13 (15.4)
Hoang et al. (2021) [50]	Penicillin	--	† (100.0)	*CTX-M-A*	0/268 (0)	2/268 (0.7)
	Ampicillin–clavulanate		† (57.8)	*CTX-M-A* and *TEM*	0/268 (0)	3/268 (1.1)
	Cefepime		† (57.3)	*CTX-M-A* and *SHV*	1/268 (0.4)	4/268 (1.5)
	Ceftriaxone		† (95.5)	*CTX-M-A*, *TEM*, and *SHV*	3/268 (1.1)	10/268 (3.7)
Ouaddane et al. (2024) [58]	Amoxicillin–clavulanate	0/1 (0)	1/1 (100)	--	--	--
	Ceftriaxone	1/1 (100)	1/1 (100)			
	Fosfomycin	1/1 (100)	0/1 (0)			
	Cotrimoxazole	1/1 (100)	1/1 (100)			
	**Antibiotic**		**Number resistant (%)**			
Alzeer et al. (1998) [19]	Amoxycillin and first-generation cephalosporin		5/5 (100.0)			
Haseeb et al. (2016) [30]	Amoxycillin–clavulanate		28/54 (52.0)			
	Ampicillin		55/59 (94.0)			
	Aztreonam		4/17 (23.5)			
	Cefazolin		8/19 (42.1)			
	Cefepime		8/30 (27.0)			
	Cefoxitin		10/50 (20.0)			
	Cefuroxime		8/23 (34.8)			
	Ceftazidime		11/46 (24.9)			
	Cefotaxime		6/26 (23.1)			
	Cephalothin		12/18 (66.7)			
	Ertapenem		3/23 (13.0)			
	Imipenem		5/6 (83.3)			
	Meropenem		3/30 (10.0)			
	Piperacillin–tazobactam		3/18 (16.7)			
	Amikacin		3/60 (5.0)			
	Gentamicin		24/63 (38.1)			
	Tobramycin		6/13 (46.2)			
	Ciprofloxacin		26/63 (41.3)			
	Levofloxacin		4/9 (44.4)			
	Moxifloxacin		16/29 (55.2)			
Marglani et al. (2016) [36]	Amoxycillin–clavulanate		6/14 (42.9)			
	Ampicillin		14/14 (100.0)			
	Imipenem		3/14 (21.4)			
	Piperacillin-tazobactam		6/14 (42.9)			

† numerator and denominator for this percentage are not available.

**Table 6:** Prevalence and acquisition of ESBL genes among pilgrims attending mass gatherings.

First author (Publication Date)	Genotypic Resistance	Pre-Hajj Number (%)	Post-Hajj Number (%)
Leangapichart et al. (2016) [32]	CTX-M genes †	13/129 (10.1)	42/129 (32.6)
	TEM genes	101/129 (78.3)	107/129 (83.0)
	SHV	82/129 (63.6)	94/129 (72.9)
Leangapichart et al. (2021) [51]	blaOXA-48	1/1 (100.0)	
	blaOXA-48-like (blaOXA-547)		1/1 (100.0)
Baharin et al. (2021) [55]	blaKPC	--	2/4 (50.0)
	blaOXA-48	--	2/4 (50.0)
Hoang et al. (2021) [50]	CTX-M-A	10/268 (3.7)	6/268 (2.2)
	TEM	2/268 (0.7)	2/268 (0.7)
	SHV	0/268 (0)	1/268 (0.4)
	CTX-M-A & TEM	3/268 (1.1)	24/268 (9.0)
	CTX-M-A & SHV	1/268 (0.4)	5/268 (1.9)
	TEM and SHV	1/268 (0.4)	1/268 (0.4)
	CTX-M-A, TEM & SHV	2/268 (0.7)	10/268 (3.7)
Ouaddane et al. (2024) [58]	ARG	260/295 (88.1)	230/291 (79.0)
	CTX-MA	68/295 (23.0)	82/291 (28.2)
	CTX-MB	30/295 (10.1)	13/291 (4.5)
	SHV	154/295 (52.0)	124/291 (42.6)
	TEM	259/295 (87.5)	218/291 (3.9)
	OXA 23	10/295 (3.9)	1/291 (0.3)
Leangapichart et al. (2017) [35]	CTX-M genes	20/218 (9.2)	71/218 (32.6)
		Acquisition rate: 68/218 (31.0%) in 2013 and 76/218 (34.8%) in 2014, overall 32.6%	
	*blaCTX-M-15*	Acquisition rate: 17/129 (13.2) in 2013 and 13/89 (14.6) in 2014	
	*blaCTX-M-78*	Acquisition rate: Overall, 7/71 (9.9)	
	*blaCTX-M-152*	Acquisition rate: Overall, 5/71 (7.0)	
	*More than one CTX-M gene acquired*	Acquisition rate: Overall, 22/218 (10.1)	

† Statistically significant difference.

**Table 7:** Antimicrobial resistance in *S. aureus* isolates identified during Hajj, Umrah, and Grand Magal de Touba.

First Author (Publication Date)	Antibiotic or Resistance Profile	Pre-Umrah (or Pre-Magal) Number (%)	Post-Umrah (or Post-Magal) Number (%)	Pre-Hajj Number (%)	Post-Hajj Number (%)
Johargy et al. (2011) [31]	MRSA	16/155 (10.3)	25/235 (10.6)	30/153 (19.6)	19/128 (14.8)
	Penicillin	136/155 (87.7)	207/235 (88.1)	132/153 (86.3)	101/128 (78.9)
	Gentamicin	4/155 (2.6)	7/235 (3.0)	14/153 (9.2)	11/128 (8.6)
	Tobramycin	6/155 (3.9)	9/235 (3.8)	18/153 (11.8)	13/128 (10.2)
	Levofloxacin	8/155 (5.2)	10/235 (4.3)	19/153 (12.4)	26/128 (20.3)
	Moxifloxacin	6/155 (3.9)	8/235 (3.4)	8/153 (5.2)	13/128 (10.2)
	Erythromycin	17/155 (11.0)	26/235 (11.1)	96/153 (62.8)	32/128 (25.0)
	Clindamycin	14/155 (9.0)	24/235 (10.2)	28/153 (18.3)	28/128 (21.9
	Tetracycline	36/155 (23.2)	54/235 (19.2)	26/153 (17.0)	63/128 (49.2)
	Fosfomycin	16/155 (10.3)	26/235 (11.1)	32/153 (20.9)	28/128 (21.9)
	Nitrofurantion	2/155 (1.3)	5/235 (2.1)	30/153 (19.6)	NR
	Fusidic Acid	16/155 (10.3)	26/235 (11.1)	NR	26/128 (20.3)
	Co-trimoxazole	3/155 (1.9)	6/235 (2.6)	22/153 (14.4)	15/128 (11.7)
	Mupirocin	1/155 (0.7)	3/235 (1.3)	12/153 (7.8)	NR
	Rifampicin	NR	NR	1/153 (0.7)	0/128 (0)
Ouaddane et al. (2024) [57] *	MRSA	22/423 (5.2)	11/423 (2.6)	--	
	mecA gene	9/140 (6.4)	6/140 (4.3)		
	mecC gene	10/102 (9.8)	4/102 (3.9)		
Dao et al. (2024) [59]	MRSA	--		25/606 (4.1)All were positive for *mecA gene*	62/606 (10.6)All positive for *mecA gene*
	**Phenotypic Resistance**		**Genotypic Resistance**		
	**Antibiotic/phenotype**	**Number (%)**	**Resistance gene**		**Number (%)**
Hoang et al. (2021) [50]	MRSA Fusidic acid Erythromycin	12/12 (100.0) 8/12 (66.7) 1/12 (8.3)	*mecA*		12/12 (100.0)
Memish et al. (2006) [38]	MRSA	6/411 (1.5)	*mecA*		6/85 (7.1)
Al-Zahrani et al. (2019) [47]	MRSA	41/89 (46.1)	SCCmec IV		21/41 (51.2)
			SCCmec V		10/41 (24.4)
			SCCmec III		5/41 (12.2)
			SCCmec II		2/41 (4.9)
			Non-Typable		3/41 (7.3)
Fatani et al. (2002) [25]	MRSA	1/47 (2.1)			
	Penicillin	38/47 (80.9)			
	Erythromycin	2/47 (4.3)			
	Cephalothin	1/4 (2.1)			
	Co-trimoxazole	2/47 (4.3)			
	Clindamycin	2/47 (4.3)			
	Tetracycline	6/47 (12.8)			
	Gentamicin	3/47 (6.4)			
Haseeb et al. (2016) [30]	***MRSA***	36/57 (63.2)			
	Amoxycillin–clavulanate	15/20 (75.0)			
	Ampicillin	17/22 (77.3)			
	Aztreonam	5/7 (71.4)			
	Cefazolin	5/6 (83.3)			
	Cefepime	3/5 (60.0)			
	Cefoxitin	3/10 (30.0)			
	Cefuroxime	4/5 (80.0)			
	Ceftazidime	4/8 (50.0)			
	Cefotaxime	3/5 (60.0)			
	Ceftriaxone	4/6 (66.7)			
	Cephalothin	13/15 (86.7)			
	Imipenem	10/20 (50)			
	Penicillin G	10/11 (90.9)			
	Gentamicin	10/24 (41.7)			
	Ciprofloxacin	15/24 (62.5)			
	Moxifloxacin	5/12 (41.7)			
	***MSSA***	21/57 (36.8)			
	Amoxycillin–clavulanate	4/14 (28.6)			
	Ampicillin	10/11 (90.9)			
	Imipenem	3/7 (42.7)			
	Oxacillin	4/17 (23.5)			
	Penicillin G	8/9 (88.9)			
	Ciprofloxacin	4/10 (40.0)			
	Moxifloxacin	4/10 (40.0)			
Marglani et al. (2016) [36]	***MRSA***	13/46 (28.2)			
	Amoxycillin-clavulanate	13/13 (100.0)			
	Ampicillin	13/13 (100.0)			
	Cefoxitin	13/13 (100.0)			
	Cefepime	13/13 (100.0)			
	Ceftazidime	13/13 (100.0)			
	Ceftriaxone	13/13 (100.0)			
	Ciprofloxacin	8/13 (61.5)			
	Levofloxacin	9/13 (69.2)			
	Gentamicin	6/13 (46.2)			
	Imipenem	13/13 (100.0)			
	Piperacillin–tazobactam	13/13 (100.0)			
	Co-trimoxazole	7/13 (53.9)			
	Clindamycin	4/13 (30.8)			
	Azithromycin	12/13 (92.3)			
	Erythromycin	10/13 (76.9)			
	Tetracycline	5/13 (38.5)			
	***MSSA***	33/46 (71.7)			
	Amoxycillin–clavulanate	7/33 (21.2)			
	Ampicillin	13/33 (39.4)			
	Ceftazidime	1/33 (3.0)			
	Ciprofloxacin	4/33 (12.1)			
	Levofloxacin	4/33 (12.1)			
	Gentamicin	1/33 (3.0)			
	Imipenem	5/33 (15.2)			
	Clindamycin	3/33 (9.1)			
	Azithromycin	7/33 (21.2)			
	Erythromycin	6/33 (18.2)			
	Tetracycline	6/33 (18.2)			
Bokhary et al. (2022) [48]	MRSA	1/121 (1.0)			

* This study refers to Grand Magal de Touba; the remaining studies in this table refer to Hajj or Umrah. NR = not reported; MRSA = methicillin-resistant *Staphylococcus aureus*; MSSA = methicillin-susceptible *Staphylococcus aureus*.

**Table 8:** Antimicrobial resistance in *S. pneumoniae* isolates identified during Hajj.

First Author (Publication Year)	Antibiotic/Resistance Profile	Pre-Hajj Number (%)	Post-Hajj Number (%)	
Ganaie et al. (2018) [27]	Co-trimoxazole	62/105 (59.0)	70/133 (52.6)	
	Tetracycline †	31/105 (29.5)	68/133 (51.1)	
	Erythromycin †	27/105 (25.7)	61/133 (45.9)	
	Levofloxacin †	6/105 (5.7)	23/133 (17.3)	
	Cefotaxime	1/105 (1.0)	0/133 (0)	
	Penicillin non-susceptible	2/105 (1.9)	1/133 (0.8)	
Memish et al. (2015) [37]	Non-susceptible to one antibiotic †	39/191 (20.4)	98/191 (51.3)	
	Non-susceptible to three antibiotics †	9/191 (4.7)	35/191 (18.3)	
Harimurti et al. (2021) [53]	Chloramphenicol	11/70 (15.7)	16/68 (23.5	
	Erythromycin	8/70 (11.4)	14/68 (20.6)	
	Clindamycin	9/70 (12.9)	8/68 (11.8)	
	Tetracycline	41/70 (58.6)	44/68 (64.7)	
	Co-trimoxazole	40/70 (57.1)	29/68 (42.6)	
	Penicillin	35/70 (50.0)	33/68 (48.5)	
	**Antibiotic/resistance profile**	**Number resistant (%)**	**Resistance gene**	**Number (%)**
Alzeer et al. (1998) [19]	Penicillin	2/6 (33.3)	NR	NR
Fatani et al. (2002) [25]	Oxacillin and Penicillin	0/24 (0)	NR	NR
Memish et al. (2016) [39]	Penicillin non-susceptible	34/110 (30.9)	*mefE* only *ermB* only *mefE* and *ermB*	12/27 (44.4) 10/27 (37.0) 5/27 (18.5)
	○ “Intermediate” susceptibility	○ 25/110 (22.7)		
	○ Resistant	○ 9/110 (8.2)		
	Amoxycillin “intermediate” susceptibility	3/110 (2.7)		
	Cefotaxime “intermediate” susceptibility	2/110 (1.8)		
	Erythromycin	27/110 (24.5)		
	Clindamycin	14/110 (12.7)		
	Tetracycline	61/110 (55.5)		
	Chloramphenicol	7/110 (6.4)		
	Co-trimoxazole			
	○ “Intermediate” susceptibility	○ 18/110 (16.4)		
	○ Resistant	○ 53/110 (48.2)		
	Levofloxacin/moxifloxacin	99/110 (0.9)		
Baharin et al. (2021) [55]	-	-	*mefA* only	5/13 (38.5)
			*pbpA* only	2/13 (15.4)
			*mefA* and *pbpA*	5/13 (38.5)
			*ermB*	0/13 (0)

† Statistically significant difference; NR = not reported.

## Supplementary Materials

The following supporting information can be downloaded at https://www.mdpi.com/article/10.3390/tropicalmed10010002/s1. Box S1: The full search strategies, including search terms.
