# Supplementary material for: Antimicrobial Resistance Associated with Mass Gatherings: A Systematic Review
Source: Trop Med Infect Dis. 2024 Dec 24;10(1):2. doi: 10.3390/tropicalmed10010002 (PMC11769525; doi:10.3390/tropicalmed10010002)
Supplement: Supplementary file 1 [file tropicalmed-10-00002-s001.zip › tropicalmed-3306296-supplementary.pdf]

Box S1: The full search strategies, including search terms

Ovid Medline

1 exp Drug Resistance, Microbial/

2 exp Anti-Bacterial Agents/

3 (anti?infect$ or anti?microb$ or anti?fung$ or anti?vir$ or anti?biotic$ or anti?bacter$).tw.

4 bacteriocid$.tw.

5 (bacteri$ or viral$ or fungal$ or microb$).tw.

6 (drug$ or treat$ or multi drug$ or multi-drug$ or pan).tw.

7 2 or 3 or 4 or 5 or 6

8 resistan$.tw.

9 7 and 8

10 1 or 9

11 exp "Anniversaries and Special Events"/

12 (mass adj2 gather$).tw.

13 exp Crowding/

14 crowd$.tw.

15 ((olympic or commonwealth or youth) and game$).tw.

16 olympiad$.tw.

17 (world adj1 cup$).tw.

18 fifa.tw.

19 europa.tw.

20 tournament$.tw.

21 (sport$ adj2 (event$ or game$ or compet$)).tw.

22 (mecca$ or makkah$).tw.

23 pilgrim$.tw.

24 (haj$ or hadj$ or hadz$).tw.

25 (umrah$ or umra$ or omra$).tw.

26 (kumbh adj1 mela).tw.

27 (world adj1 youth adj1 day$).tw.

28 ((music$ or religio$ or cultural$) adj2 festival$).tw.

29 11 or 12 or 13 or 14 or 15 or 16 or 17 or 18 or 19 or 20 or 21 or 22 or 23 or 24 or 25 or 26 or 27 or 28

30 10 and 29

31 exp animals/ not humans.sh.

32 30 not 31

Ovid Embase

1 exp antibiotic resistance/

2 exp antiinfective agent/

3 (anti?infect$ or anti?microb$ or anti?fung$ or anti?vir$ or anti?biotic$ or anti?bacter$).tw.

4 bacteriocid$.tw.

5 (bacteri$ or viral$ or fungal$ or microb$).tw.

6 (drug$ or treat$ or multi drug$ or multi-drug$ or pan).tw.

7 2 or 3 or 4 or 5 or 6

8 resistan$.tw.

9 7 and 8

10 1 or 9

11 (mass adj2 gather$).tw.

12 exp "crowding (area)"/

13 crowd$.tw.

14 exp "sports and sport related phenomena"/

15 ((olympic or commonwealth or youth) and game$).tw.

16 olympiad$.tw.

17 (world adj1 cup$).tw.

18 fifa.tw.

19 europa.tw.

20 tournament$.tw.

21 (sport$ adj2 (event$ or game$ or compet$)).tw.

22 (mecca$ or makkah$).tw.

23 pilgrim$.tw.

24 (haj$ or hadj$ or hadz$).tw.

25 (umrah$ or umra$ or omra$).tw.

26 (kumbh adj1 mela).tw.

27 (world adj1 youth adj1 day$).tw.

28 ((music$ or religio$ or cultural$) adj2 festival$).tw.

29 11 or 12 or 13 or 14 or 15 or 16 or 17 or 18 or 19 or 20 or 21 or 22 or 23 or 24 or 25 or 26 or 27 or 28

30 10 and 29

31 exp animal/ not human/

32 30 not 31

Cochrane Library

ID Search Hits
#1 MeSH descriptor: [Drug Resistance, Microbial] explode all trees
#2 MeSH descriptor: [Anti-Bacterial Agents] explode all trees
#3 (anti-infect* OR anti-microb* OR anti-fung* OR anti-vir* OR antibiotic* OR anti-bacter*):ti,ab,kw
#4 bacteriocid*:ti,ab,kw
#5 (bacteri* OR viral* OR fungal* OR microb*):ti,ab,kw
#6 (drug* OR treat* OR multi drug* OR multi-drug* OR pan):ti,ab,kw
#7 #2 OR #3 OR #4 OR #5 OR #6
#8 resistan*:ti,ab,kw
#9 #7 AND #8
#10 #1 OR #9
#11 MeSH descriptor: [Anniversaries and Special Events] explode all trees
#12 (mass NEAR/2 gather*):ti,ab,kw
#13 MeSH descriptor: [Crowding] explode all trees
#14 crowd*:ti,ab,kw
#15 ((olympic OR commonwealth OR youth) AND game*):ti,ab,kw
#16 olympiad*:ti,ab,kw
#17 (world NEAR/1 cup*):ti,ab,kw
#18 fifa:ti,ab,kw
#19 europa:ti,ab,kw
#20 tournament*:ti,ab,kw
#21 (sport* NEAR/2 (event* OR game* OR compet*)):ti,ab,kw
#22 (mecca* OR makkah*):ti,ab,kw
#23 pilgrim*:ti,ab,kw
#24 (haj* OR hadj* OR hadz*):ti,ab,kw
#25 (umrah* OR umra* OR omra*):ti,ab,kw
#26 (kumbh NEAR/1 mela):ti,ab,kw
#27 (world NEAR/1 youth NEAR/1 day*):ti,ab,kw
#28 ((music* OR religio* OR cultural*) NEAR/2 festival*):ti,ab,kw
#29 #11 OR #12 OR #13 OR #14 OR #15 OR #16 OR #17 OR #18 OR #19 OR #20 OR #21 OR #22 OR #23 OR #24 OR #25 OR #26 OR #27 OR #28
#30 #10 AND #29

SCOPUS

(((TITLE-ABS-KEY (anti-infect* OR anti-microb* OR anti-fung* OR anti-vir* OR antibiotic* OR anti-bacter*)) OR (TITLE-ABS-KEY (bacteriocid*)) OR (TITLE-ABS-KEY (bacteri* OR viral* OR fungal* OR microb*)) OR (TITLE-ABS-KEY (drug* OR treat* OR "multi drug*" OR multi-drug* OR

pan))) AND (TITLE-ABS-KEY (resistan*))) AND ((TITLE-ABS-KEY (((music* OR religio* OR cultural*) W/2 festival*))) OR (TITLE-ABS-KEY (world W/1 youth W/1 day*)) OR (TITLE-ABS-KEY (kumbh W/1 mela)) OR (TITLE-ABS-KEY (umrah* OR umra* OR omra*)) OR (TITLE-ABS-KEY (haj* OR hadj* OR hadz*)) OR (TITLE-ABS-KEY (pilgrim*)) OR (TITLE-ABS-KEY (mecca* OR makkah*)) OR (TITLE-ABS-KEY ((sport* W/2 (event* OR game* OR compet*)))) OR (TITLE-ABS-KEY (tournament*)) OR (TITLE-ABS-KEY (europa)) OR (TITLE-ABS-KEY (fifa)) OR (TITLE-ABS-KEY (world W/1 cup*)) OR (TITLE-ABS-KEY (olympiad*)) OR (TITLE-ABS-KEY (((olympic OR commonwealth OR youth) AND game*))) OR (TITLE-ABS-KEY (crowd*)) OR (TITLE-ABS-KEY (mass W/2 gather*))) AND NOT ((INDEXTERMS (animals OR animal)) AND NOT (INDEXTERMS (humans OR human)))
